# Causal inference and evidence-based recommendations in occupational health and safety research

**DOI:** 10.5271/sjweh.3929

**Published:** 2020-10-30

**Authors:** Reiner Rugulies, Alex Burdorf

**Affiliations:** National Research Centre for the Working Environment Copenhagen, Denmark; Department of Public Health and Department of of Psychology, University of Copenhagen, Copenhagen, Denmark [e-mail: rer@nfa.dk]; Department of Public Health, Erasmus Medical Centre, Rotterdam, The Netherlands [e-mail: a.burdorf@erasmuscmc.nl]

In this issue of the Journal, a group of distinguished Nordic researchers, led by Anne Helene Garde and including four of our Associated Editors, present a discussion paper that originated from a workshop and provides detailed recommendations on night shift work (1). The recommendations are very clear: to protect workers’ health, night shift schedules should have: (i) ≤3 consecutive night shifts; (ii) shift intervals of ≥11 hours; and (iii) ≤9 hours shift duration. For pregnant women, night work should be limited to one shift per week. The authors acknowledge that under circumstances allowing better possibilities for daytime sleep, recommendations could be different.

The discussion paper is remarkable in that it provides clear and strong recommendations based on what the authors themselves call a “limited literature”, thus a limited scientific evidence on the risk of shift work for cancer and other health and safety risks. In a recent editorial, British researchers concluded that, due to heterogeneity of shift working in longitudinal studies, it is too difficult to draw a firm conclusion about the risk of breast cancer, let alone about an exposure threshold for night shift work (2). Yet, both Nordic and British researchers seemed to agree that we should not postpone recommendations on best practice in shift work scheduling for reasons of lack of certainty on causal inference.

For the most important health and safety conditions the Nordic authors are concerned with – cancer, cardiovascular disease, diabetes, injuries and pregnancy-related outcomes – the evidence relies on observational studies. Although longitudinal studies on shift work increasingly use register-based exposure information on working hours patterns, often based on payroll data that is linked with registers in healthcare (3, 4), these studies are still vulnerable to important biases, such as selection bias and residual confounding. There are several examples in the literature of well-conducted observational studies suggesting an effect of an exposure that subsequently was not corroborated in randomized controlled trials (RCT). One of the most famous examples is hormone replacement therapy (HRT) in post-menopausal women. Numerous observational studies suggested a protected effect of HRT with regard to risk of cardiovascular disease (5, 6), but when an RCT was finally conducted, it found the effect of HRT to be more harmful than beneficial (7). Recently, a large-scale RCT found no effect of vitamin D intake on reduced risk of depression (8), despite numerous observational studies suggesting such an effect (9). Thus, there are good reasons to treat results from observational studies with caution.

On the other hand, exercising caution does not mean that one should abstain from making recommendations when evidence is based on observational studies only, in the hope that this would keep one on the safe side of scientific scrutiny. There is no safe side. In accordance with Paul Watzlawick’s famous quote that “one cannot not communicate” (10), it can be reasoned that not making recommendations is also a form of recommendation, the recommendation to continue business as usual. The recommendation to stop asbestos production, which rather came too late than too early, was not based on RCT but observational studies on the multiple health-hazardous effects of asbestos (11). Thus, when considering the evidence, researchers should not only consider the best evidence based on available data and their causal inference, but also the potential consequences of continuing current practice.

Fifty-five years ago, Sir Austin Bradford Hill published his famous nine viewpoints on causal inference in health research (12). As pointed out by Bradford Hill, as well as other scholars (13), none of the nine viewpoints (today mostly known as “criteria”) ensures that an observed observation is causal, however, they still might be helpful in assessing the confidence whether or not a measure of association indicates a causal link between two variables. Today, causal inference remains an intensively discussed topic. In its December 2016 issue, the *International Journal of Epidemiology* published a series of articles, discussion papers and letters on causal inference in epidemiology, in particular on the merits and limitations of the counterfactual “potential outcome approach”, which relies heavily on experiments whether induced by the researcher or natural changes in particular situations that may be interpreted as happening at random (14, 15). This approach has been criticized by proponents of a more “pluralistic approach” for a variety of reasons, among others that it limits causality to particular factors that are usually not widely generalizable (16, 17). Very recently (September 2020), in an opinion paper (18), the main proponent of the potential outcome approach, Tyler VanderWeele asked: “Can sophisticated study designs with regression analyses of observational data provide causal inferences?” The answer seem to be a cautious “yes”. Regarding single observational studies, VanderWeele lists eight considerations that increase confidence in the estimate, including longitudinal design; the quality of the assessment of exposure, outcome and confounders; flexible statistical modeling examining robustness to modelling decisions; and attempts to address unmeasured confounding. Evidence then may evolve from accumulation of results from multiple high-quality studies, in particular if these have different designs that are subject to different biases (18).

The struggle on causal interpretation and subsequent evidence-based recommendations is also visible in the GRADE (Grading of Recommendations, Assessments and Evaluations) system, which rates the certainty of evidence and the strengths of recommendations in systematic reviews, for example in *Scandinavian Journal of Work, Environment and Health* articles (19-22). As GRADE has its origins in healthcare evaluation, its evidence assessment favors the RCT, and although the GRADE working group encourages applying GRADE to observational studies (23), the quality rating of observational studies always starts with “low quality”, with possibilities for upgrading and downgrading, whereas the quality rating of RCT starts with “high quality”. The recently developed “Navigation Guide” (24) - a methodology for synthesizing evidence in systematic reviews that evolved from environmental research but is now also applied in occupational health research (25, 26) - recommends a different approach, where the quality assessment of observational studies starts with “moderate” before the process of up- or downgrading (24).

The paper by Garde et al (1) is not a systematic review, it uses neither GRADE nor Navigation Guide methodology and does not grade the evidence. It is a discussion paper written by leading researchers in the field that base their conclusions and recommendations on their knowledge of the literature, including systematic reviews. Given that a substantial proportion of the workforce is exposed to some type of night shift work, this is a bold, but necessary, step. We are looking forward to further research, both original studies and reviews, corroborating or challenging the conclusions and recommendations of this discussion paper.
